# Cor Pulmonale Secondary to Severe Pulmonary Hypertension As the First Manifestation of Graves' Disease

**DOI:** 10.7759/cureus.28115

**Published:** 2022-08-17

**Authors:** Sandra D Santos, David Lopes Sousa, Ana Cristina Martins, João Rua, Pedro Ribeiro

**Affiliations:** 1 Internal Medicine, Centro Hospitalar e Universitário de Coimbra, Coimbra, PRT; 2 Intensive Care Unit, Centro Hospitalar e Universitário de Coimbra, Coimbra, PRT

**Keywords:** trabs, quality of life, heart failure, graves' disease, pulmonary hypertension

## Abstract

Pulmonary hypertension (PH), especially if severe, carries a significant morbidity and mortality. Herein we describe a case of an 88-year-old woman with severe heart failure and several hospitalizations that year for the same reason, rapid re-admission after discharge and loss of walking ability. Transthoracic echocardiography (TTE) revealed severe pulmonary hypertension (PASP=69 mmHg) and right ventricular dysfunction without left structural or functional dysfunction. Pulmonary thromboembolism, relevant pulmonary pathology was excluded, and an extended autoimmune study was also negative. Thyroid disorders were investigated, a Graves' disease with thyrotoxicosis was diagnosed and promptly treated with thiamazole, also known as methimazole. There was a rapid improvement in the clinical and hemodynamic status of the patient, stabilization of the right heart failure (HF), resolution of the volume overload and a TTE showed improvement with moderate PH (PASP=55 mmHg). This case shows a reversible cause of PH and highlights how treatment of Graves' disease can reduce pulmonary artery pressure and contribute to symptomatic relief and better quality of life.

## Introduction

Pulmonary hypertension (PH) is a severe, multifactorial, multi-causal clinical syndrome that compromises physical capacity and causes great morbidity. It consists of an increase in pulmonary arterial pressure by a progressive increase in pulmonary vascular resistance, which tends to evolve with right ventricular failure and premature death of the patient. Thyroid disorders have been reported to be associated with several cardiovascular diseases, namely pulmonary hypertension. We report a case of Graves’ disease associated with severe pulmonary hypertension with adequate treatment having a positive impact in pulmonary artery pressure and symptom relief.

This article was previously presented as a poster to the 18th European Congress of Internal Medicine, Lisbon, Portugal, 29-31 August, 2019.

## Case presentation

**Figure 1:**
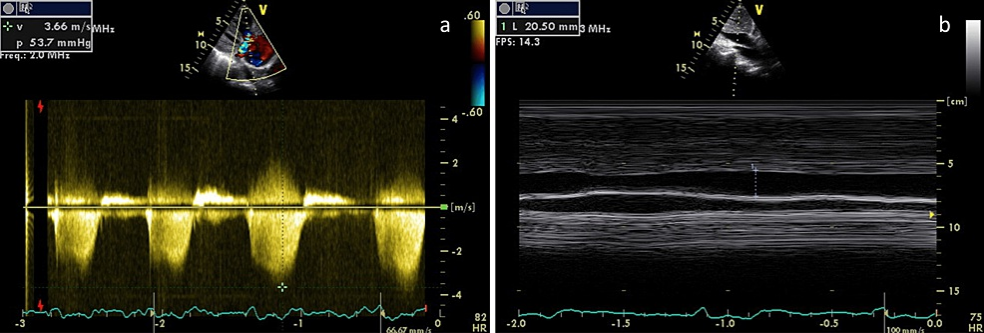
Estimated pulmonary arterial systolic pressure before Graves' treatment Echocardiographic parameters, before Graves’ treatment, showing estimated pulmonary arterial systolic pressure of 69 mmHg. Systolic artery pressure was estimated from a) tricuspid regurgitant jet velocity of 3.66 m/s and calculated by Bernoulli equation maximum gradient of 54 mmHg plus b) estimated right atrial pressure of 15 mmHg (slightly dilated inferior vena cava with respiratory variation < 50%).

An 88-year-old woman, with a history of chronic kidney disease stage IIIa, atrial fibrillation, heart failure (HF) and hypertension, was evaluated at the hospital because of dyspnea and edema of the lower limbs. She was hemodynamically stable, apyretic, with absence of murmur in both lung bases, with hepatomegaly, ascites and marked lower limbs edema. She had type 1 respiratory insufficiency, atrial fibrillation with rapid ventricular rate, and increased Nt-proBNP (20,000 pg/ml). The patient was diagnosed with severe heart failure and was hospitalized. There was a history of two similar hospitalizations that year, with rapid re-admission after discharge and loss of walking ability. Three days later, she presented acute pulmonary edema and underwent a transthoracic echocardiogram (TTE), which showed severe PH [pulmonary arterial systolic pressure (PASP) 69 mmHg; (Figure 1)] and right ventricular dysfunction without left structural or functional dysfunction. An AngioCT excluded pulmonary thromboembolism.

The PH investigation was initiated, although the right catheterization was refused, including a CT-CTAP that excluded relevant pulmonary pathology and an extended, also negative, autoimmune study. Thyroid function and thyrotropin receptor antibody (TRAbs) evaluation revealed a Graves' disease with thyrotoxicosis (Table 1).

**Table 1:** Thyroid Tests TSH: Thyroid-stimulating hormone; T4: Thyroxine; T3: Triiodothyronine; TRAbs: TSH receptor autoantibodies

	Before Treatment	One Month After treatment	Normal Range
TSH (IU/ml)	<0.008	0.037	0.4-4.0
T4 (ng/dl)	3.2	1.6	0.8-1.6
T3 (pg/ml)	3.7	3	1.8-4.2
TRAbs (IU/ml)	1.6	1.1	<1.0

**Figure 2:**
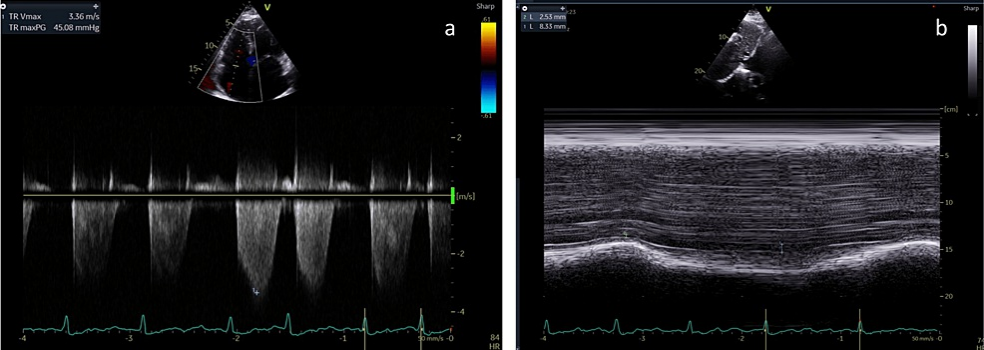
Estimated pulmonary arterial systolic after Graves' treatment Echocardiographic parameters, evaluated after Graves’ treatment and TRAbs levels decrease, showing an estimated pulmonary arterial systolic of 55 mmHg. Systolic artery pressure was estimated from a) tricuspid regurgitant jet velocity of 3.36 m/s and calculated by Bernoulli equation maximum gradient of 45 mmHg plus b) estimated right atrial pressure of 10 mmHg (slightly dilated inferior vena cava with respiratory variation > 50%).

Treatment with propylthiouracil, 100 mg three times a day, was started and later replaced with thiamazole due to an allergic reaction. After four weeks of treatment with thiamazole, Free T4 levels were within the normal range of values (Table 1) and there was a rapid improvement in the clinical and hemodynamic status of the patient, stabilization of the right HF and resolution of the volume overload. A new TTE showed marked improvement with moderate PH (PASP 55 mmHg; Figure 2).

At discharge, the patient was hemodynamically stable, with no need for oxygen therapy or relevant limitation of physical activity (NYHA I) and was able to walk. At one month reevaluation appointment, the patient remained stable.

## Discussion

We report a case of a patient who had pulmonary hypertension associated with Graves' disease. The patient had a clear clinical course of right HF due to severe PH. We excluded other causes of PH and observed an improvement in pulmonary artery pressure after Graves' disease treatment, which allowed the identification of Graves' disease as the underlying cause of pulmonary hypertension in this case.

Pulmonary hypertension is a progressive and fatal disease, characterized by abnormal elevation of pressure in the pulmonary artery, with progressive pulmonary vascular resistance. It has multiple etiologies; the World Health Organization Group 5, the group with unclear multifactorial mechanisms, includes patients with thyroid disorders [1]. Patients with PH have both pulmonary vasoconstriction and vascular remodeling that includes smooth-muscle and endothelial proliferation, in situ thrombosis and the formation of plexiform lesions [1]. Pulmonary hypertension has been increasingly described to be associated with hyperthyroidism or other types of thyrotoxicosis. Some reports indicate a high prevalence of autoimmune thyroid disease in patients with pulmonary hypertension. The exact mechanism is still unknown, but an autoimmune process associated with endothelial damage or dysfunction, increased cardiac output resulting in endothelial injury, and increased metabolism of intrinsic pulmonary vasodilating substances, have been considered [2].

Hyperthyroidism is an important, but often neglected, cause of PH. An abnormal increase of circulating thyroid hormone has a close relationship with target organ damage, including cardiovascular complications. Elevated levels of thyroid hormone can directly act on myocardium and exert positive chronotropic and inotropic effect by enhancing cardiac excitability and myocardial contraction. In addition to direct effect, high concentrations of thyroid hormone could also stimulate angiogenesis.

Furthermore, Sugiura et al. [3] found a direct correlation between elevated pulmonary artery systolic pressure and TRAbs, which are antibodies that lead to an increase in the amount of thyroid hormones, through the stimulation of the thyroid cellular receptor. These authors hypothesized that TRAbs could indirectly influence the development of PH by inducing an immune-mediated damage to the endothelium which could promote endothelial cell dysfunction and hyperproliferation, leading to PH development.

Importantly, medical treatment of hyperthyroidism and TRAbs levels reduction can significantly reduce pulmonary arterial pressure and improve the outcomes. The case here reported is an example of this, since the successful treatment of Graves' led to a significant reduction of pulmonary arterial pressures and to a relevant improvement in the patient quality of life, with few hospitalizations. Such effect has also been observed in other patients in whom treatment of Graves' disease led to nearly complete normalization of elevated pulmonary artery pressure and symptomatic relief [2-5].

## Conclusions

The evidence from this case report gives additional support to the observation that Graves' disease can present in association with PH. Thyroid disorders should be considered in the differential diagnosis of patients with pulmonary hypertension, because the treatment of the underlying thyroid disease can lead to a reduction of pulmonary arterial pressure, potentially avoiding the side effects and costs of current therapies for pulmonary hypertension and limiting the consequences of untreated thyroid disorders.
